# Investigation of Women's Knowledge and Beliefs About Cervical Cancer and Cervical Smear Examination: A Survey Study in Greece

**DOI:** 10.7759/cureus.75486

**Published:** 2024-12-10

**Authors:** Sophia Kontonikou, Giannoula Kyrkou, Anastasia Bothou, Christina Nanou, Victoria Vivilaki, Anna Deltsidou

**Affiliations:** 1 Outpatient Clinics, Chalastra Health Centre, Thessaloniki, GRC; 2 Department of Midwifery, Univesrity of West Attica, Athens, GRC; 3 Department of Midwifery, University of West Attica, Athens, GRC

**Keywords:** cervical cancer, hpv, human papillomavirus, pap smear, screening

## Abstract

Aim

This study aimed to investigate women's knowledge and beliefs about cervical cancer and cervical smear examinations.

Methodology

The research was conducted at a health center in Greece from May 15, 2023, to August 15, 2023. The study sample consisted of 160 women aged 21-65 years who attended the health center. The data were collected by using a questionnaire. The first part of the questionnaire was based on the Cervical Cancer Awareness and Symptoms Initiative (CCASI) questionnaire, while the second part used the Health Belief Model questionnaire for cervical cancer and the Champion Pap Test.

Results

A total of 157 participants (98.1%) reported having a Pap test at some point in their lives, and 18 (11.25%) of them had a pathological result. Many women had better knowledge about the factors that increase the risk of cervical cancer. Most participants (118, 73.8%) knew that there was a cervical cancer control program in Greece and 150 (93.8%) were aware of the HPV vaccine. Age and education were independently associated with awareness of the cervical cancer screening program in Greece.

Conclusions

The findings of this study indicate a positive attitude of women toward cervical cancer prevention. The level of knowledge about cervical cancer and the Papanicolaou test among the women who visited the health center was sufficient; however, there were several areas where they lacked knowledge about symptoms and risk factors. The study underlines the importance of implementing preventive measures among women for the effective fight against cervical cancer.

## Introduction

According to the World Health Organization (WHO), cervical cancer is the fourth most common cancer in women. It is responsible for approximately 604,127 new cases and 341,831 deaths per year, worldwide [1]. Eighty-seven percent of deaths occur in third-world countries [2], with a higher mortality rate in sub-Saharan Africa [3]. Developed countries have seen a significant reduction in the incidence and mortality from invasive cancer over the past 50 years due to mass screening [4]. Globally, the median age of diagnosis of the disease is 53 years [5].

The human papillomavirus (HPV), particularly the HPV16 and HPV18 subtypes, is the primary risk factor for cervical cancer [6]. Other risk factors include having sex at an early age, smoking, having multiple sexual partners, taking oral contraceptive pills for a very long time, and certain sexually transmitted diseases (STDs) [7]. Women infected with HIV are more likely to develop cervical cancer than women who are not infected with HIV [8].

Primary (HPV vaccination) and secondary (screening) preventive strategies can significantly lower the risk of cervical cancer [9]. Reducing HPV infection is the fundamental public health goal of cervical cancer primary prevention. Biological processes, such as HPV vaccination, and behavior modification techniques can be used for primary prevention [5]. Secondary prevention involves the implementation of various measures aimed at early detection and effective management of cervical cancer. These measures primarily include screening for cervical cancer and effective management of precancerous lesions [10]. This can be achieved by various methods, such as the Pap test, the visual inspection of the cervix after acetic acid application (VIA) and/or of Lugol's iodine (VILI), and the HPV test for detecting high-risk HPV types.

Aim

This study aimed to investigate the knowledge and beliefs of women attending a health center in Greece about cervical cancer and cervical smear examinations.

The objective of this study was to interview women who do not live in urban areas and to investigate whether they know about cervical cancer, its causes, risk factors, about cervical smear examinations, including their purpose, frequency, and how they are performed. Moreover, to explore what are women's attitudes towards cervical cancer screening, whether they believe screening is important, and their beliefs about the effectiveness of screening in preventing cervical cancer. Finally, to investigate if their socioeconomic status could affect the results.

These objectives can gain a better understanding of women's knowledge, attitudes, and beliefs about cervical cancer and cervical smear examinations. This information can be used to develop effective strategies to increase screening rates and ultimately reduce the burden of cervical cancer.

## Materials and methods

Study setting design

This is a cross-sectional quantitative study, and data collection was conducted by completing a specially designed questionnaire given to the participants to complete themselves. The sample of this study consisted of 160 women who were followed up at the public outpatient gynecological clinics of the Pyrgos “Chalastra” Health Center, Greece, from May 15, 2023, to August 15, 2023.

The inclusion and exclusion criteria of this study are given in Table 1.

**Table 1:** Inclusion and exclusion criteria.

Inclusion criteria
Women aged 18-65 years old
Women residing in villages around the Health Center of Chalastra
Women who are fluent in Greek
Women who are mentally competent to provide informed consent
Women who are willing to participate in the study and complete all study procedures
Exclusion criteria
Women who have undergone a hysterectomy
Pregnant women
Women who are unable to provide informed consent due to cognitive impairment or other reasons

A specially designed questionnaire was used. The first part of the questionnaire was based on the Cervical Cancer Awareness and Symptoms Initiative (CCASI) questionnaire and the primary Cancer Awareness Measures (CAM) questionnaire, developed by Cancer Research UK, University College London, King's College London, and Oxford University in 2007-2008 [11]. This questionnaire was developed by UCL Health Behavior Research in collaboration with the Department of Health Cancer Team and Eve Appeal [12]. This questionnaire included 29 questions that identify women's ability to identify symptoms, warning signs, and risk factors associated with cervical cancer and explore their knowledge of screening programs and the availability of the HPV vaccine in the country (Appendices A-B).

The second part of the questionnaire was the Health Belief Model questionnaire for cervical cancer and the Champion's Pap Smear Test, as applied in the study by Guvenc et al. [13]. Thirty-six questions were formulated, with participants responding based on their level of agreement using a five-point Likert-type scale. The scale included the following options: (1) Strongly disagree, (2) Disagree, (3) Neither disagree nor agree, (4) Agree, and (5) Strongly agree. The questions addressed women's beliefs about the severity of risk and their susceptibility to cervical cancer, health factors, and the benefits and barriers to women having a Pap test.

The translation of the two questionnaires was completed through a multi-stage process by three bilingual translators according to official adaptation guidelines proposed by the European Organisation for Research and Treatment of Cancer (EORTC) for translation and validation of scales [14]. Initially, they were translated from English into Greek by two translators, whose native language was Greek. The translators were obstetricians-gynecologists with expertise in gynecological health problems and an excellent level of English. A reverse translation was then conducted, from Greek to English, by a third translator who was not affiliated with the profession or gynecological health issues. The third translator was proficient in the language. Finally, a meeting was held among the translators to confirm the semantic, conceptual, and experiential equivalence of the two questionnaires, the original English version and the translated Greek version.

The third part of the questionnaire captured the basic sociodemographic characteristics of women such as age, marital status, number of children, level of education, and professional status. Participants were also asked to indicate whether they had a cancer patient among their family or friends; whether they smoked, and if so, how many cigarettes they smoked per day; whether they were menopausal; whether they had ever undergone a Pap test; whether they had ever received an abnormal Pap test result; and whether they had undergone any treatment in response to an abnormal result.

Ethical considerations

Both survey questionnaires adhere strictly to the code of ethics and behavior. Participants' personal information was kept private, their anonymity was preserved, and participation in the study was entirely voluntary. The research undertaken satisfied all national and international ethical and moral requirements, including the Helsinki Convention. Following the General Data Protection Regulation's (GDPR's) guidelines, personal data were handled and processed (EU 2018/1725). Ethical approval was given by the Research Ethics Committee of the University of West Attica (Reference Number 48320, May 17, 2023).

Statistical analysis

The Kolmogorov-Smirnov test was used to assess the normality of the distributions of the quantitative variables. While the means and standard deviations (SDs) were used to define the regularly distributed data, the median and interquartile range were also used to characterize the non-normally distributed data. To characterize the qualitative variables, absolute (*N*) and relative (%) frequencies were employed.

Spearman's correlation coefficient was used to test the relationship between two quantitative variables. Linear regression analysis was used to find independent factors associated with participants' knowledge of cervical cancer warning signs and risk factors, as well as the dimensions of the cervical cancer health scale and Pap test, which yielded dependence coefficients (β) and their standard errors (SEs).

When the distribution of the dependent variable was not normal, the logarithm of the dependent variable was used in the linear regression. To identify independent variables linked to knowledge of the Greek cervical cancer screening program, vaccination awareness, and appropriate Pap test screening, logistic regression analysis was carried out. The results included odds ratios (ORs) along with their 95% confidence intervals (95% CIs). The significance levels were two-sided, with statistical significance set at 0.05. The analysis was performed using the SPSS statistical software, version 26.0 (IBM Corp., Armonk, NY).

## Results

The sample consisted of 160 women who attended a health center in Greece, with a mean age of 46.1 years (SD = 10.0 years). Of the 160 women, 120 (75%) were married and 86 (53.7%) had two children. Also, 83 (51.9%) were higher education graduates and 88 (55%) were employed full-time (Table 2).

**Table 2:** Sociodemographic data of study participants.

		N	%
Marital status	Single	16	10
	Married	120	75
	Divorced	17	10.6
	Widowed	6	3.8
	Cohabitation agreement	1	0.6
Number of children	None	27	16.9
	One	25	15.6
	Two	86	53.7
	Three	19	11.9
	Four and more	3	1.9
Educational level	Illiterate	2	1.2
	Primary education	14	8.8
	Secondary education	61	38.1
	Tertiary education	83	51.9
Working status	Full-time employed	88	55.0
	Employed part-time	10	6.2
	Unemployed	14	8.8
	Retired	11	6.9
	Self-employed	17	10.6
	Student	2	1.3
	Household	17	10.6
	Disabled or too ill to work	1	0.6

Of the 160 participants, 3 (1.9%) had either cancer themselves or a partner with cancer. Regarding smoking, 61 (38.1%) of them gave a positive response and 24 (39.3%) smoked 5-10 cigarettes per day.

Fifty-five (34.4%) participants were menopausal, and the median time of the last menstrual period was 50 years (range 48-50 years). A total of 157 participants (98.1%) had a Pap test, and 134 (85.4%) had regular Pap tests. For 113 participants (72%) who had a Pap test, the most recent test was within the past year, while 18 (11.5%) had an abnormal Pap test at some point, and all followed treatment (Table 3).

**Table 3:** Participants’ gynecological history. SD, standard deviation

		N	%
Are you in menopause?	No	105	65.6
	Yes	55	34.4
If yes, when did you have the last period (age) Mean Value (SD) Median		49.2 (3.1)	50.0 (48.0-50.0)
Did you have a Pap test?	No	3	1.9
	Yes	157	98.1
If yes:	Once	3	1.9
	2 to 3 times	20	12.7
	Regular (every year for over than five years)	134	85.4
When was the last time?	In the last year	113	72.0
	Before 1-3 years	40	25.5
	≥3 years	4	2.5
Have you ever had a pathological Pap test ?	No	139	88.5
	Yes	18	11.5
If yes, have you had any treatment?	No	0	0.0
	Yes	18	100

Of the 160 participants, 92 (57.5%) knew that vaginal bleeding between periods could be a sign of cervical cancer, only 14 (8.8%) of them thought that persistent back pain could also be a sign of cervical cancer, and 65 (40.6%) knew that a persistent vaginal discharge that smells unpleasant is also a sign.

As signs of cervical cancer, 75 participants (46.9%) correctly identified discomfort or pain during sexual intercourse, 67 (41.9%) identified bleeding during menstruation that is more intense or lasts longer than usual, 15 (9.4%) identified persistent diarrhea, 109 (68.1%) identified vaginal bleeding after menopause, 74 (46.3%) identified persistent pelvic pain, 90 (56.3%) identified vaginal bleeding during or after sexual intercourse, 29 (18.1%) identified blood in stool or urine, and 80 (50%) identified unexplained weight loss (Table 4).

**Table 4:** Warning signs for cervical cancer.

The following may or may not be warning signs of cervical cancer. We are interested in your opinion:		N	%
Do you think vaginal bleeding between periods could be a sign of cervical cancer?	No	21	13.1
	Yes	92	57.5
	I do not know	47	29.4
Do you think persistent back pain could be a sign of cervical cancer?	No	84	52.5
	Yes	14	8.8
	I do not know	62	38.8
Do you think a persistent vaginal discharge that smells unpleasant could be a sign of cervical cancer?	No	55	34.6
	Yes	65	40.6
	I do not know	39	24.5
Do you think discomfort or pain during sexual intercourse could be a sign of cervical cancer?	No	39	2.,5
	Yes	75	46.9
	I do not know	45	28.3
Do you believe that bleeding during your period that is heavier or lasts longer than usual could be a sign of cervical cancer?	No	47	29.6
	Yes	67	41.9
	I do not know	45	28.3
Do you think persistent diarrhea could be a sign of cervical cancer?	No	84	52.5
	Yes	15	9.4
	I do not know	61	38.1
Do you think vaginal bleeding after menopause could be a sign of cervical cancer?	No	13	8.1
	Yes	109	68.1
	I do not know	38	23.8
Do you think persistent pelvic pain could be a sign of cervical cancer?	No	22	13.8
	Yes	74	46.3
	I do not know	64	40.0
Do you think vaginal bleeding during or after sexual intercourse could be a sign of cervical cancer?	No	18	11.3
	Yes	90	56.3
	I do not know	52	32.5
Do you think blood in the stool or could it be a sign of cervical cancer in the urine?	No	79	49.7
	Yes	29	18.1
	I do not know	51	32.1
Do you think unexplained weight loss could be a sign of cervical cancer?	No	35	2.9
	Yes	80	50.0
	I do not know	45	28.1

The average of the right responses to the questions regarding the symptoms of cervical cancer was then multiplied by 100 to get a knowledge score. The values of 1 and 0 were awarded to the correct and incorrect replies, respectively. A higher score suggested a greater understanding of the symptoms of cervical cancer. The score in this sample ranged from 0 to 100 points with a mean of 40.4 points (SD = 22.8 points). The Cronbach's α reliability coefficient was greater than 0.7, indicating acceptable reliability (Table 5).

**Table 5:** Knowledge score regarding signs of cervical cancer. SD, standard deviation

	Least value	Higher value	Mean value (SD)	Μedian (intermediate range)	Cronbach’s α
Knowledge of the symptoms of cervical cancer	0.0	100.0	40.4 (22.8)	45.5 (18.2-54.6)	0.72

The majority of participants (154, 96.3%) would go to the doctor immediately if they had a symptom of cervical cancer. One participant (0.6%) said they did not know, another 1 (0.6%) said they would go within the month, and 4 (2.5%) said they would not go soon (Table 6).

**Table 6:** Responses from study participants regarding how quickly they would see their doctor if they had any symptoms of cervical cancer.

	N	%
Immediately	154	96.3
I do not know	1	0.6
Within the month	1	0.6
Not soon	4	2.5

Of the 160 participants, 95 (59.4%) believed that cervical cancer is independent of age, 40 (25%) thought it is more likely for women aged 30-49, 18 (11.2%) thought it is more likely for those aged 50-69, 4 (2.5%) thought it is more likely for those over 70, and only 3 (1.9%) thought it would be more likely for women in the 20-29 age group (Table 7).

**Table 7:** Responses of study participants regarding the question, Within the next year, who is most likely to develop cervical cancer in Greece?

Within the next year, who is most likely to develop cervical cancer in Greece?	N	%
A woman aged 20-29 years	3	1.9
A woman aged 30-49 years	40	25.0
A woman aged 50-69 years	18	11.3
A woman aged 70 and more	4	2.5
Cervical cancer is independent of age	95	59.4

Of the 160 participants, 106 (66.3%) correctly agreed that HPV infection increases the risk of cervical cancer, while 86 (53.8%) correctly disagreed that non-smokers have a higher risk of cervical cancer. Also, the risk of cervical cancer was known by 109 (68.1%) to be increased by a weakened immune system, 61 (38.1%) by long-term use of a contraceptive pill, 76 (47.5%) by chlamydia infection, 12 (7.5%) by having an uncircumcised sexual partner, and 44 (27.5%) by having sexual intercourse at a young age. Of the 160 participants, 20 (12.5%) correctly agreed that the risk is increased by having several children, 74 (46.3%) agreed that it is increased by having a sexual partner who has had multiple previous sexual partners, and 143 (89.4%) agreed that it is increased by not having regular Pap tests (Table 8).

**Table 8:** Percentages of agreement among study participants about how much certain sociodemographic characteristics or medical history elements can increase a woman's likelihood of developing cervical cancer. HPV, human papillomavirus

The following may or may not increase a woman's chance of developing cervical cancer. How much do you agree that each of these may increase a woman's chance of developing cervical cancer?		N	%
Infection with the HPV	I disagree strongly/Disagree	4	2.5
	I am not sure	50	31.3
	Agree/Strongly agree	106	66.3
Non-smoker	I disagree strongly/Disagree	86	53.8
	I am not sure	39	24.4
	Agree/Strongly agree	35	21.9
Existence of a weakened immune system (e.g., due to HIV/AIDS, immunosuppressive drugs, or transplantation)	I disagree strongly/Disagree	7	4.4
	I am not sure	44	27.5
	Agree/Strongly agree	109	68.1
Long-term use of the contraceptive pill	I disagree strongly/Disagree	21	13.1
	I am not sure	78	48.8
	Agree/Strongly agree	61	38.1
Chlamydia infection	I disagree strongly/Disagree	13	8.1
	I am not sure	71	44.4
	Agree/Strongly agree	76	47.5
Having a sex partner who is not circumcised	I disagree strongly/Disagree	82	51.3
	I am not sure	66	41.3
	Agree/Strongly agree	12	7.5
I am starting sexual intercourse at a young age (before 17 years).	I disagree strongly/Disagree	53	33.1
	I am not sure	63	39.4
	Agree/Strongly agree	44	27.5
Having multiple sexual partners	I disagree strongly/Disagree	32	20.0
	I am not sure	31	19.4
	Agree/Strongly agree	97	60.6
Existence of several children	I disagree strongly/Disagree	87	54.4
	I am not sure	53	33.1
	Agree/Strongly agree	20	12.5
Having a sexual partner who has had several previous sexual partners	I disagree strongly/Disagree	47	29.4
	I am not sure	39	24.4
	Agree/Strongly agree	74	46.3
Not conducting regular Pap tests	I disagree strongly/Disagree	10	6.3
	I am not sure	7	4.4
	Agree/Strongly agree	143	89.4

A knowledge score on factors that increase the risk of cervical cancer was then calculated as the average of the correct answers multiplied by one hundred. Correct answers were assigned a value of 1 and incorrect answers were assigned a value of 0. Higher scores implied better knowledge about factors that increase the risk of cervical cancer. The score in this sample ranged from 0 to 90.9 points, with a mean of 47.1 points (SD = 19.9 points). The Cronbach's α reliability coefficient was greater than 0.7, indicating acceptable reliability (Table 9).

**Table 9:** Score of responses from study participants regarding their knowledge of factors that increase the risk of cervical cancer. SD, standard deviation

	Least value	Higher value	Mean value (SD)	Μedian (intermediate range)	Cronbach’s α
Knowledge of factors that increase the risk of cervical cancer	0.0	90.9	47.1 (19.9)	45.5 (36.4-63.6)	0.71

In the study, 42 (26.3%) of the 160 participants were moderately to very confident that they would notice a symptom of cervical cancer. Of the 160 participants, 118 (73.8%) were aware of the cervical cancer screening program in Greece, and 150 (93.8%) were aware of the HPV vaccine (Table 10).

**Table 10:** Confidence of study participants in observing symptoms of cervical cancer, knowledge of its screening program in Greece, and the vaccine that protects against its occurrence.

		N	%
How confident are you that you would notice a symptom of cervical cancer?	Not at all sure	26	16.3
	Not much sure	82	51.3
	Sure enough	42	26.3
	Very sure	10	6.3
As far as you know, is there a cervical cancer screening program in Greece?	No	4	2.5
	Yes	118	73.8
	I don’t know	38	23.8
As far as you know, is there a vaccine that protects against cervical cancer in Greece?	No	0	0.0
	Yes	150	93.8
	I don’t know	10	6.3

Knowledge scores on cervical cancer risk factors were significantly and positively correlated (*P *< 0.001). Therefore, better knowledge about the risk-increasing factors for cervical cancer implies better knowledge about its warning signs. There was no significant association between knowledge scores and health scale beliefs about cervical cancer and Pap test (Table 11).

**Table 11:** Knowledge scores on cervical cancer warning signs, risk factors, and dimensions of the cervical cancer health scale and the Pap test. Spearman's rho. *P* represents *P*-value. The statistical significance of the *P*-value was set at 0.05.

		Knowledge of the risk factors for cervical cancer	Knowledge of the warning signs of cervical cancer
Knowledge about the risk factors for cervical cancer	rho		0.8
	P		<0.001
Benefits and motivations of the Pap test	rho	0.09	0.14
	P	0.259	0.082
Barriers to Pap testing	rho	0.08	-0.09
	P	0.328	0.274
Perceived severity of cervical cancer	rho	0.02	0.12
	P	0.791	0.122
Cervical cancer susceptibility	rho	0.02	0.09
	P	0.834	0.279
Motivation of health	rho	0.01	0.11
	P	0.987	0.153

Multivariate linear regressions were performed to find factors independently associated with knowledge of cervical cancer warning signs and risk factors for increasing the risk of cervical cancer, with the dependent variable being the score on these scores and the independent variables being the women's demographics and gynecological history. The results of the analyses are given in Table 12. Only marital status was found to be independently associated with knowledge of cervical cancer warning signs (*P *= 0.012). Those who were married had more knowledge of the warning signs compared to those who were not. Age was found to be independently associated with knowledge of cervical cancer risk factors (*P *= 0.047). Older age implies more knowledge of cervical cancer risk factors (Table 12).

**Table 12:** Correlation of study participants' "sociodemographic and gynecological history" with dependent variable "knowledge score about cervical cancer warning signs" and knowledge score on knowledge of cervical cancer risk factors". ^+^Dependence coefficient. Note: The logarithm of the dependent variable has been used. ^++^Standard error. ^+++^Standardized coefficient. ^*^P-value: The statistical significance of the *P*-value was set at 0.05.

Dependent variable knowledge score about cervical cancer warning signs			β+	SE++	b+++	*P*-value*
Age			-0.004	0.006	-0.089	0.492
Married (Yes vs. no)			0.248	0.098	0.213	0.012*
Number of children			-0.088	0.050	-0.167	0.080
Education		Secondary vs. up to primary	-0.191	0.140	-0.186	0.174
		Tertiary education vs. up to primary	-0.059	0.146	-0.059	0.685
Employed (Yes vs. no)			0.160	0.087	0.156	0.069
Smoking(Yes vs. no)			0.124	0.120	0.118	0.305
Are you in menopause? (Yes vs. no)			-0.204	0.296	-0.056	0.492
Dependent variable knowledge score on knowledge of cervical cancer risk factors						
Age			0.010	0.004	0.269	0.047*
Married (Yes vs. no)			-0.047	0.062	-0.067	0.448
Number of children			0.001	0.032	0.004	0.966
Education	Secondary vs. up to primary		0.002	0.090	0.002	0.987
	Tertiary education vs. up to primary		0.033	0.093	0.054	0.726
Employed (Yes vs. no)			0.015	0.056	0.024	0.788
Smoking (Yes vs. no)			-0.131	0.077	-0.205	0.091
Are youin menopause? (Yes vs. no)			-0.036	0.189	-0.016	0.851

Then, to find factors independently associated with the dimensions of the cervical cancer health scale and the Pap test, multivariate linear regressions were performed with the score on these dimensions as the dependent variable and the women's demographics and gynecological history as independent variables.

Menopause (*P *= 0.037) and marital status (*P *= 0.05) were found to be independently correlated with the score on *Benefits and motivation from the Pap test*. Those women who were in menopause gave less importance to benefits and motivation from the Pap test compared to those who were not, while married women gave more importance compared to unmarried women (Table 13).

**Table 13:** Multivariate linear regression with dependent variables being the score on the dimension "Benefits and motivation from the Pap test" and "Obstacles to Pap test" and the independent variables being "the demographic data of the women and their gynecological history." Note: The logarithm of the dependent variable has been used. ^+^Dependence coefficient. ^++^Standard error. ^+++^Standardized coefficient. ^*^P-value: The statistical significance of the *P*-value was set at 0.05.

		β+	SE++	b+++	*P*-value*
Dependent variable *Benefits and motivation from the Pap test*					
Age		0.001	0.001	0.229	0.080
Married(Yes vs. no)		0.026	0.009	0.239	0.050*
Number of children		-0.002	0.005	-0.050	0.606
Education	Secondary vs. up to primary	0.000	0.013	-0.002	0.991
	Tertiary education vs. up to primary	0.009	0.014	0.101	0.496
Employed(Yes vs. no)		-0.012	0.008	-0.130	0.134
Smoking(Yes vs. no)		-0.011	0.008	-0.115	0.153
Are you in menopause? (Yes vs. no)		-0.024	0.011	-0.247	0.037*
Dependent variable *Obstacles to Pap test*					
Age		0.001	0.002	0.046	0.715
Married (Yes vs. no)		-0.030	0.026	-0.097	0.240
Number of children		0.003	0.013	0.018	0.846
Education	Secondary vs. up to primary	-0.018	0.037	-0.065	0.627
	Tertiary Education vs. up to primary	-0.088	0.038	-0.330	0.023*
Employed(Yes vs. no)		-0.051	0.013	-0.252	0.050*
Smoking(Yes vs. no)		0.009	0.021	0.033	0.670
Are you in menopause? (Yes vs. no)		-0.009	0.032	-0.031	0.786

Educational level and work status were found to be independently associated with barriers to Pap test performance (*P *= 0.05). Specifically, higher education graduates faced fewer barriers compared to those with up to primary education (*P *= 0.023) and female workers faced fewer barriers compared to those who were unemployed. The educational level had the greatest effect on facing barriers, followed by work status (Table 13).

Subsequently, to find factors independently associated with women's awareness of the cervical cancer screening program and the vaccine, multivariate logistic regressions were performed with awareness of the screening program and the HPV vaccine as dependent variables and the demographics of the participating women as independent variables. The results of the analyses are given in Table 14. Age (*P *= 0.016) and education (*P *= 0.036) were found to be independently associated with awareness of the cervical cancer screening program in Greece. Specifically, older age was associated with a greater likelihood that women were aware of the screening program and those who were secondary school graduates were 4.04 times more likely to know about the screening program compared to those who were up to primary school graduates. Similarly, tertiary school graduates were 4.67 times more likely to know about the screening program compared to primary school graduates (*P *= 0.027) (Table 14).

**Table 14:** Multivariate logistic regression with dependent variable "information about the screening program in Greece" and "the demographic data of the participating women" as the independent variables and with the dependent variable "being the correct screening in Greece" and independent variables being the study participants' "knowledge scores of cervical cancer warning signs, cervical cancer risk factors, cervical cancer health scale, and Pap test and vaccine awareness." OR, odds ratio *P-value: The statistical significance of the *P*-value was set at 0.05.

		OR	*P*-value*
Dependent variable *Information about the screening program in Greece*			
Age		1.08 (1.01-1.14)	0.016
Number of children		1.18 (0.73-1.9)	0.503
Married (Yes vs. no)		0.58 (0.21-1.56)	0.280
Education	Secondary vs. up to primary	4.04 (1.1-14.9)	0.036*
	Tertiary education vs. up to primary	4.67 (1.2-18.27)	0.027*
Employed (Yes vs. no)		1.09 (0.47-2.51)	0.845
Are you in menopause? (Yes vs. no)		1.01 (0.3-3.38)	0.984
Dependent variable *Being the correct screening in Greece*			
Knowledge of the signs of cervical cancer		0.99 (0.96-1.01)	0.328
Knowledge of cervical cancer risk factors		1.01 (0.98-1.03)	0.674
Benefits and motivation from Pap test		2.61 (0.75-9.09)	0.132
Barriers to Pap test		0.16 (0.06-0.43)	<0.001*
Perceived severity of cervical cancer		0.62 (0.25-1.57)	0.316
Sensitivity to cervical cancer		0.81 (0.31-2.12)	0.668
To the extent you know, is there a vaccine that protects against cervical cancer in Greece?		1.11 (0.2-6.1)	0.908

Multivariate logistic regression was performed to find the factors independently associated with proper screening, with proper screening as the dependent variable and independent knowledge scores of cervical cancer warning signs, its risk increasing factors, with the dimensions of the cervical cancer health scale and Pap test and vaccine awareness dimensions (Table 14). Barriers to Pap smear testing were found to be independently associated with proper screening with Pap smear testing (*P *< 0.001). Increased barriers faced by women were associated with a decreased likelihood of correct screening with a Pap test.

## Discussion

The results of this study showed that a high percentage of the participants had been tested with the Pap test. More specifically, 157 participants (98.1%) stated that they had undergone a Pap test. Among them, 134 (85.4%) reported having it regularly (every year for more than five years), four (2.5%) had their last test more than three years ago, and only three (1.9%) had never undergone one. In a similar study conducted in Arkhangelsk, Northwest Russia, only 37.1% of the participants had undergone cervical cytology less than three years ago, 7% had their last test more than three years ago, and 38% had never had a Pap test [15].

The findings also suggest that the level of knowledge is satisfactory for both risk factors and symptoms. Specifically, 68.1% of women recognized vaginal bleeding after menopause as a symptom of cervical cancer, followed by 92 women (57.5%) who identified vaginal bleeding between menstrual periods and 90 women (56.3%) who identified vaginal bleeding during or after sexual intercourse. In contrast, persistent back pain had the lowest proportion (14, 8.8%). In a similar study conducted in Malta, 74.94% of participants were able to identify more than three symptoms when asked, with the most recognized symptoms being postmenopausal bleeding, persistent pelvic pain, and weight loss [16]. While in a study conducted in India, menstrual bleeding (30.75%) and smelly vaginal discharge (28.86%) were the most common symptoms reported [17].

A satisfactory proportion of women seem to be aware of the risk factors associated with cervical cancer. Most of the sample (106 participants, 66.3%) correctly agreed that HPV infection increases the risk of cervical cancer, 44 participants (27.5%) identified initiating sexual intercourse at a young age as a risk factor, and 74 participants (46.3%) recognized that having a sexual partner with multiple previous partners increases the risk of cervical cancer. Data from a similar study conducted in India show that 32.68% of women knew that the early age of marriage was a risk factor for cervical cancer followed by 23.01% of women who reported that the early age of initiation of sexual activity was a common risk factor [17]. Also, in a similar study conducted in Malta, unfortunately, only 38.1% of the surveyed population knew that HPV infection was a risk factor [16].

As this study found, women with better knowledge of the risk factors for cervical cancer also had greater knowledge of its warning signs. Moreover, married women had more knowledge of the warning signs, and older women had more knowledge of cervical cancer risk factors. In a similar study conducted in Fasa, Southern Iran, participants with higher educational levels had higher knowledge. They performed Pap tests more frequently than those with lower educational levels [18]. A similar study conducted in Johannesburg found that a higher level of knowledge about cervical cancer and screening increased women's likelihood of having a Pap test [19].

The results of women's knowledge about the HPV vaccine are very encouraging. Most women (150, 93.8%) were informed about the vaccine. Similar results were observed in a survey conducted in Greece, which included a study sample of 1,000 parents of adolescent girls; 98.8% of the participants were aware of the existence of this vaccine [20]. In another study conducted in Malta, 56.27% of the participants knew the vaccination schedule and half of them knew the correct age of administration of the vaccine [16].

According to a survey of Hispanic women in the state of Indiana, older women and those with higher educational levels were more likely to follow the recommendations for cervical cancer screening, while single women and those with higher incomes were less likely to regularly screen for the disease [21]. In the present study, older women were more likely to be aware of the screening program. Also, those who were secondary school graduates were 4.04 times more likely to know about the screening program compared to those who were primary school graduates. Similarly, tertiary school graduates were 4.67 times more likely to know about the screening program compared to those up to primary school graduates.

In terms of barriers to having a Pap test, a study in the United Kingdom found that time, pain, discomfort, and embarrassment were the most reported barriers, as were difficulty making appointments and fear of the results [22]. The lack of time is because most women invited for screening are working and have multiple family commitments. A study conducted in Johannesburg found that women who perceive more barriers to accessing a Pap test are less likely to have had one. The most commonly cited barriers were fear of abnormal results, shame, and the perception that the Pap test is painful and time-consuming [19].

In addition, according to the findings of a study conducted in Turkey, women with low socioeconomic status (education, employment, and income) had low scores on health motivational barriers and high scores on perceived barriers. The most significant barrier to the Pap test was found to be the male gender of the health care professional (53.9). Barrier scores were high in younger women, large families, and women who were stillborn [23]. It is worth mentioning that in the present study, higher education graduates and working women faced fewer barriers to performing the Pap test compared to those who had completed up to primary education or were unemployed. In addition, this study found that menopausal women placed less importance on the benefits and motivation for the Pap test compared to non-menopausal women, while married women placed more importance compared to unmarried women.

Finally, this study found that those women who smoked showed less susceptibility to future cervical cancer than those who did not smoke. In another study conducted in Fasa, Southern Iran, it was found that non-smoking women had significantly more knowledge about the Pap test [18].

This study enabled small populations in remote villages in northern Greece to participate in a scientific study regarding knowledge and beliefs about cervical cancer and taking the Pap smear, for the first time. Also, our study was able to explore the relationship between knowledge and beliefs and actual screening and vaccination behavior. Based on the results of this study, educational interventions can be designed to improve knowledge.

The limitations of this study were the small number of participants from a small area of ​​northern Greece, which means that the results cannot be generalized to other populations. Also, knowledge and beliefs may be subject to social desirability bias, which means that participants may not always give accurate or truthful answers.

## Conclusions

In conclusion, the findings of this study provide valuable information about the knowledge and beliefs of the women in the sample about cervical cancer. The women who participated in the study showed a positive attitude toward cervical cancer prevention.

The establishment of a Pap test and HPV vaccine information hotline is one idea for boosting participation in cervical cancer screening. Information campaigns should be created by health policymakers as well. The goal of the information campaigns is to increase knowledge about HPV vaccination and cervical cancer to aid in the early detection of symptoms. Women within the age range for whom screening is recommended should be the primary audience for these ads, with a particular focus on those from disadvantaged socioeconomic backgrounds and lower education levels. Finally, the dissemination of these crucial messages can also be aided using electronic media, including internet pages, television commercials, and online videos.

## Appendices

Appendix A 

**Table 15:** Demographic_ Questionnaire

We would now like to ask you a few questions about yourself. This will help us analyze the results of the survey. The data collected will help us identify specific age groups or groups of people with specific demographic characteristics who need more information about cervical cancer. Your name will not be asked and all your answers will be kept strictly confidential and anonymous. Your data will be stored under the Data Protection Act. Your information will not affect your medical care in any way.				
How old are you?	Write your age (number)			
	I prefer not to answer			
What is your marital status?	Single			
	Married/I stay with my partner			
	Married but separated			
	Divorced			
	Widow			
	Cohabitation agreement			
	I prefer not to answer			
How many children do you have?	None			
	One			
	Two			
	Three			
	Four or over than			
	I prefer not to answer			
What is your educational status?	Illiterate			
	Primary education			
	Secondary education			
	Trietrty education (Technological / University)			
Professional status	Fulltime job			
	Part-time job			
	Unemployed			
	Freelancer			
	Retired			
	Householder			
	Student			
	Disabilities or too ill (I am not able to work)			
	I prefer not to answer			
Do you, your family, or close friends have cancer?	Yes	No	I do not Know	I prefer not to answer
You				
Partner				
A close member of the family				
Another member of the family				
Close friend				
Another friend				
Do you smoke?				
If yes, how many cigarettes do you smoke?	Until 5 cigarettes			
	5-10 cigarettes			
	10-15 cigarettes			
	15-25 cigarettes			
	More than 25 cigarettes			
Are you in menopause?	Yes			
	No			
If yes, when was your last period?	Yes			
	No			
Did you have any Pap test?	Yes			
	No			
If yes:	Once			
	2 to 3 times			
	Regular (every year for over 5 years)			
When was the last time?	In the year			
	1 to 3 years ago			
	Over 3 years ago			
Have you ever had a pathological Pap test?	Yes			
	No			
If so, did you undergo any treatment?	Yes			
	No			
Thank you for your time!!!				

Appendix B 

**Table 16:** Questionnaire

QUESTIONNAIRE								
The following may or may not be warning signs of cervical cancer. We are interested in your opinion:				Yes		No		I don’t know
Do you think vaginal bleeding between periods could be a sign of cervical cancer?								
Do you think persistent back pain could be a sign of cervical cancer?								
Do you think a persistent vaginal discharge that smells unpleasant could be a sign of cervical cancer?								
Do you think discomfort or pain during sexual intercourse could be a sign of cervical cancer?								
Do you think that bleeding during your period that is heavier or lasts longer than usual could be a sign of cervical cancer?								
Do you think persistent diarrhea could be a sign of cervical cancer?								
Do you think vaginal bleeding after menopause could be a sign of cervical cancer?								
Do you think persistent pelvic pain could be a sign of cervical cancer?								
Do you think vaginal bleeding during or after sexual intercourse could be a sign of cervical cancer?								
Do you think blood in the stool or could it be a sign of cervical cancer in the urine?								
Do you think unexplained weight loss could be a sign of cervical cancer?								
If you had a symptom that you thought might be a sign of cervical cancer, how soon would you contact your doctor to make an appointment to discuss it?								
Within the next year, who is most likely to develop cervical cancer in Greece?								
A woman aged 20 to 29 years old								
A woman aged 30 to 49 years old								
A woman aged 50 to 69 years old								
A woman aged 70 and over								
Cervical cancer is independent of age								
The following may or may not increase a woman's chance of developing cervical cancer. How much do you agree that each of these may increase a woman's chance of developing cervical cancer?	I disagree strongly	Disagree	I am not sure		Agree		Strongly agree	
Infection with the HPV virus (Human Papillomavirus)								
Non-smoker								
Existence of a weakened immune system (e.g. due to HIV/AIDS, immunosuppressive drugs, or transplantation)								
Long-term use of the contraceptive pill								
Chlamydia infection								
Having a sex partner who is not circumcised								
I am starting sexual intercourse at a young age (before 17 years).								
Having multiple sexual partners								
Existence of several children								
Having a sexual partner who has had several previous sexual partners								
Not conducting regular Pap tests								
How confident are you that you would notice a symptom of cervical cancer?	Not at all sure							
	Not much sure							
	Sure enough							
	Very sure							
As far as you know, is there a cervical cancer screening program in Greece?	Yes							
	No							
	I don’t know							
If yes, at what age are women invited to have their first Pap test to detect cervical cancer?								
As far as you know, is there a vaccine that protects against cervical cancer in Greece?	Yes							
	No							
	I don’t know							
If yes, at what age is it offered?								
Please give just ONE answer	I disagree strongly	Disagree	I neither disagree nor agree		Agree		Strongly agree	
1. I am likely to get cervical cancer in the future								
2. My chances of getting cervical cancer in the next few years are high								
3. I think I will get cervical cancer at some point in my life								
4. The thought of cervical cancer scares me								
5. When I think of cervical cancer, my heart beats faster								
6. I'm afraid to think about cervical cancer								
7. If I get cervical cancer, the problems I experience will last for a long time								
8. Cervical cancer would threaten my relationship with my partner								
9. If I got cervical cancer my whole life would change								
10. If I had cervical cancer, I would not live more than 5 years								
11. I want to identify health problems early								
12. Maintaining good health is extremely important to me								
13. I am looking for information on how to improve my health								
14. I think it is important to engage in activities that improve my health								
15. I eat good and balanced meals								
16. Exercise at least 3 times a week								
17. I have regular medical check-ups, even if I'm not sick								
18. If I have a Pap test regularly and the results are good, I should not be too worried about cervical cancer								
19. Pap test will detect changes in the cervix before they become cancer								
20. Even if cervical cancer was detected in a Pap test, it was not that difficult to treat								
21. I think that regular Pap test (Pap test) is the best way to diagnose cervical cancer								
22. Regular screening with a Pap test (Pap test) will reduce my chances of dying from cervical cancer								
23. I am afraid to do a Pap test in case the results are not good								
24. I am afraid to take a Pap test because I don't know what will happen								
25. I don't know where to go to do the Pap test (Pap test)								
26. I would be embarrassed to lie on the gynecological examination bed showing private parts of my body to do the Pap test								
27. Pap test takes a long time								
28. Pap test causes very severe pain								
29. Health professionals who do the Pap test are very rude to women								
30. I neglect or forget to do the Pap test (Pap test) regularly								
31. I have other problems more important than doing the Pap test								
32. I am too old to have a regular Pap test								
33. There is no health center or other government health facility near my home to do the Pap test								
34. If I am fated to get cervical cancer, the Pap test will not prevent it								
35. I prefer a female doctor or midwife to do the Pap test								
36. I am not going to have a Pap test if I have to pay for it								
Thank for your time!!!								
